# Biomedical implications from a morphoelastic continuum model for the simulation of contracture formation in skin grafts that cover excised burns

**DOI:** 10.1007/s10237-017-0881-y

**Published:** 2017-02-08

**Authors:** Daniël C. Koppenol, Fred J. Vermolen

**Affiliations:** Delft Institute of Applied Mathematics, Delft University of Technology, Delft, The Netherlands

**Keywords:** Burns, Skin graft contraction, Contracture formation, Splinting therapy, Tissue remodeling, Morphoelasticity, Biomechanics, Moving-grid finite-element method, Element resolution refinement / recoarsement, Flux-corrected transport (FCT) limiter, 35L65, 35M10, 65C20, 68U20, 74L15, 92C10, 92C17

## Abstract

A continuum hypothesis-based model is developed for the simulation of the (long term) contraction of skin grafts that cover excised burns in order to obtain suggestions regarding the ideal length of splinting therapy and when to start with this therapy such that the therapy is effective optimally. Tissue is modeled as an isotropic, heterogeneous, morphoelastic solid. With respect to the constituents of the tissue, we selected the following constituents as primary model components: fibroblasts, myofibroblasts, collagen molecules, and a generic signaling molecule. Good agreement is demonstrated with respect to the evolution over time of the surface area of unmeshed skin grafts that cover excised burns between outcomes of computer simulations obtained in this study and scar assessment data gathered previously in a clinical study. Based on the simulation results, we suggest that the optimal point in time to start with splinting therapy is directly after placement of the skin graft on its recipient bed. Furthermore, we suggest that it is desirable to continue with splinting therapy until the concentration of the signaling molecules in the grafted area has become negligible such that the formation of contractures can be prevented. We conclude this study with a presentation of some alternative ideas on how to diminish the degree of contracture formation that are not based on a mechanical intervention, and a discussion about how the presented model can be adjusted.

## Introduction

In the United Kingdom, approximately 250,000 citizens get injured due to burning each year (Hettiaratchy and Dziewulski 2004). In the United States, about half a million citizens require medical treatment as a result of thermal injury each year (Gibran et al. 2013). The majority of these burns are minor and do not require specialized care. However, a small portion of the injuries is extensive and as a consequence roughly 13,000 individuals in the United Kingdom and approximately 40,000 individuals in the United States, are admitted to a hospital or burn center for treatment each year (Gibran et al. 2013; Hettiaratchy and Dziewulski 2004).

The core treatment of burns in these medical centers consists usually of two subparts; first most of the burnt skin is excised surgically and thereafter the newly created wound is covered by a skin graft. The use of a skin graft to cover a newly created wound has two widely recognized benefits compared to the situation where these wounds are left to heal by secondary intention alone; in general it reduces both the overall contraction of the grafted area and the development of hypertrophic scar tissue in these areas (Walden et al. 2000). Unfortunately, however, many times skin grafts still contract considerably after placement on their recipient bed and this may result then in substantial shrinkage of the grafts and hence the development of contractures in these tissues (Kraemer et al. 1988).

The development of contractures is a serious complication that has a significant impact on an affected person’s quality of life, and often requires substantial further corrective surgery (Leblebici et al. 2006). Therefore, therapies have been developed which aim at the prevention of the formation of contractures. The main therapy in current usage focuses on counteracting the mechanical forces generated within the contracting graft by means of static splinting of the covered wound after placement of the graft (Richard and Ward 2005). How effective splinting therapy is in preventing contracture formation is actually unclear at the moment; it is a fact that contracture formation is still a common complication despite the frequent application of splinting therapy (Schouten et al. 2012). This could be a consequence of the fact that it is unclear at present what the optimal point in time is after placement of the skin graft to start with the therapy. Furthermore, it could also be a consequence of the fact that it is also unclear how long the static splints have to be worn for the therapy to be effective.

This unsatisfactory situation is probably partly caused by the fact that actually little is known about the etiology of the formation of contractures (Harrison and MacNeil 2008). We think that a better understanding of the mechanism underlying contracture formation probably aids in the development of a better treatment plan that reduces the development of this sequela, and argue that computational modeling studies can contribute to the expansion of this understanding. Therefore we develop here a new mathematical model for the simulation of the contraction of skin grafts that cover excised burns in order to gain new insights into the mechanism underlying the formation of contractures. Based on the obtained insights, we give suggestions regarding the ideal length of splinting therapy and when to start with the therapy such that the therapy is effective optimally. In addition, we put forward some alternative ideas on how to diminish the degree of contracture formation that are not based on a mechanical intervention.

The development of the model is presented in Sect. 2. Subsequently, the simulation results are presented in Sect. 3. Here, we also show good agreement with respect to the evolution over time of the surface area of unmeshed skin grafts that cover excised burns between outcomes of computer simulations obtained in this study and scar assessment data gathered previously in a clinical study. The model and the simulation results are discussed in Sect. 4.

## Development of the mathematical model

Given that contraction mainly takes place in the dermal layer of skin tissues, we incorporate solely a portion of this layer into the model. The layer is modeled as a heterogeneous, isotropic, morphoelastic continuous solid with a modulus of elasticity that is dependent on the local concentration of the collagen molecules. With respect to the mechanical component of the model, the displacement of the dermal layer ($\mathbf {u}$), the displacement velocity of the dermal layer ($\mathbf {v}$), and the infinitesimal effective strain present in the dermal layer ($\mathbf {\varepsilon }$) are chosen as the primary model variables (The latter variable represents a local measure for the difference between the current configuration of the dermal layer and a hypothetical configuration of the dermal layer where the tissue is mechanically relaxed (See also Eq. (3))). Furthermore, we select the following four constituents of the dermal layer as primary model variables: fibroblasts (*N*), myofibroblasts (*M*), a generic signaling molecule (*c*), and collagen molecules ($\rho$).

In order to incorporate the formation of contractures (i.e., the formation of long term deformations) into the model, we use the theory of morphoelasticity developed by Hall (2009). Central to this theory is the assumption that the classical deformation gradient tensor (i.e., $\mathbf {F}$) can be decomposed into a product of two tensors (i.e., $\mathbf {F} = \mathbf {A}\mathbf {Z}$) (Hall 2009; Goriely and Ben Amar 2007; Rodriguez et al. 1994). The tensor $\mathbf {Z}$ can be thought of as the locally defined deformation from the fixed reference configuration to a hypothetical configuration (i.e., a zero stress state (Fung 1993)) wherein the internal stresses around all individual points in the dermal layer are relieved, and the tensor $\mathbf {A}$ can be thought of as the locally defined deformation from this hypothetical configuration to the current configuration of the dermal layer. Based on this decomposition, Hall derived several related evolution equations that describe mathematically the change of the effective strain over time. Hence, these equations basically give a mathematical description of the remodeling of the dermal layer over time. In this study we assume that the effective strains are small. Therefore, we use here the evolution equation that describes the dynamic change of the infinitesimal effective strain over time (i.e., Eq. (1c) in this study and Eq. (5.64) in the PhD thesis of Hall (2009)). (The derivation of this evolution equation is rather lengthy and contains numerous subtleties. Therefore, we present here solely the finally derived equation. The full derivation of the evolution equation can be found in the PhD thesis of Hall.)

Combined with the general conservation equations for mass and linear momentum in local form, the following continuum hypothesis-based framework is used as basis for the model:

1a $$\begin{aligned}&\frac{\text {D} z_{i}}{\text {D} t} + z_{i}\left[ \nabla \cdot \mathbf {v}\right] = -\nabla \cdot \mathbf {J}_{i} + R_{i}, \end{aligned}$$

1b $$\begin{aligned}&\frac{\text {D}(\rho _{t}\mathbf {v})}{\text {D} t} + \rho _{t}\mathbf {v}\left[ \nabla \cdot \mathbf {v}\right] = \nabla \cdot \mathbf {\sigma } + \mathbf {f}, \end{aligned}$$

1c $$\begin{aligned}&\frac{\mathcal {D}\mathbf {\varepsilon }}{\mathcal {D}t} + [\text {tr}(\mathbf {\varepsilon }) - 1]\text {sym}(\mathbf {L}) = - \mathbf {G}, \end{aligned}$$

where

2 $$\begin{aligned} \mathbf {v}= & {} \frac{\text {D}\mathbf {u}}{\text {D} t}, \end{aligned}$$

3 $$\begin{aligned} \mathbf {\varepsilon }= & {} \mathbf {I} - \mathbf {A}^{-1}, \end{aligned}$$

and

4 $$\begin{aligned} \frac{\mathcal {D}\mathbf {\varepsilon }}{\mathcal {D}t} = \frac{\text {D}\mathbf {\varepsilon }}{\text {D}t} + \mathbf {\varepsilon }\text {skw}(\mathbf {L}) - \text {skw}(\mathbf {L})\mathbf {\varepsilon }. \end{aligned}$$

Equation (1a) is the conservation equation for the cell density / concentration of constituent *i* of the dermal layer, Eq. (1b) is the conservation equation for the linear momentum of the dermal layer, and Eq. (1c) is the evolution equation that describes how the infinitesimal effective strain changes over time. Within the above equations $z_{i}$ represents the cell density / concentration of constituent *i*, $\mathbf {J}_{i}$ represents the flux associated with constituent *i* per unit area due to random dispersal, chemotaxis and other possible fluxes, $R_{i}$ represents the chemical kinetics associated with constituent *i*, $\rho _{t}$ represents the total mass density of the dermal tissues, $\mathbf {\sigma }$ represents the Cauchy stress tensor associated with the dermal layer, $\mathbf {f}$ represents the total body force working on the dermal layer, $\mathbf {L}$ is the displacement velocity gradient tensor (i.e., $\mathbf {L} = \nabla \mathbf {v}$), and $\mathbf {G}$ is a tensor that describes the rate of active change of the effective strain. The operator $\mathcal {D}(\cdot )/\mathcal {D}t$ is the Jaumann time derivative, and the operator $\text {D}(\cdot )/\text {D}t$ is the material time derivative. (If the material time derivative is applied to the effective strain tensor, then it is applied to each of the scalar elements of this tensor separately.) Given the chosen primary model variables, we have $i \in \{N,M,c,\rho \}$. In order to simplify the notation somewhat we replace $z_{i}$ by *i* in the remainder of this study. Hence, $z_{N}$ becomes *N*, $z_{M}$ becomes *M*, and so on.

### The cell populations

The functional forms for the biochemical kinetics associated with the (myo)fibroblasts and the functional forms for the movement of these cells are identical to functional forms used previously (Koppenol et al. 2017b). For the biochemical kinetics, the functional forms are

5 $$\begin{aligned} R_{N}= & {} r_{F}\left[ 1 + \frac{r_{F}^{\max }c}{a_{c}^{I} + c}\right] [1 - \kappa _{F}F]N^{1 + q}\nonumber \\&-\, k_{F}cN - \delta _{N}N, \end{aligned}$$

6 $$\begin{aligned} R_{M}= & {} r_{F}\left\{ \frac{\left[ 1 + r_{F}^{\max }\right] c}{a_{c}^{I} + c}\right\} [1 - \kappa _{F}F]M^{1 + q}\nonumber \\&+\, k_{F}cN - \delta _{M}M, \end{aligned}$$

where

7 $$\begin{aligned} F = N + M. \end{aligned}$$

The parameter $r_{F}$ is the cell division rate, $r_{F}^{\max }$ is the maximum factor with which the cell division rate can be enhanced due to the presence of the signaling molecule, $a_{c}^{I}$ is the concentration of the signaling molecule that causes the half-maximum enhancement of the cell division rate, $\kappa _{F}F$ represents the reduction in the cell division rate due to crowding, *q* is a fixed constant, $k_{F}$ is the signaling molecule-dependent cell differentiation rate of fibroblasts into myofibroblasts, $\delta _{N}$ is the apoptosis rate of fibroblasts, and $\delta _{M}$ is the apoptosis rate of myofibroblasts. The functional forms for the cell fluxes are

8 $$\begin{aligned} \mathbf {J}_{N}= & {} -D_{F}F\nabla N + \chi _{F}N\nabla c, \end{aligned}$$

9 $$\begin{aligned} \mathbf {J}_{M}= & {} -D_{F}F\nabla M + \chi _{F}M\nabla c, \end{aligned}$$

where $D_{F}$ is the cell density-dependent random motility coefficient of the (myo)fibroblasts, and $\chi _{F}$ is the chemotactic coefficient.

### The generic signaling molecule

The functional form for the net production of the generic signaling molecule (i.e., the first term on the right hand side of Eq. (10)) and the functional form for the dispersion of the signaling molecule are identical to functional forms used previously (Koppenol et al. 2017b):

10 $$\begin{aligned} R_{c}= & {} k_{c}\left[ \frac{c}{a_{c}^{II} + c}\right] \left[ N + \eta ^{I} M\right] - \delta _{c}g(N,M,c,\rho )c, \end{aligned}$$

11 $$\begin{aligned} \mathbf {J}_{c}= & {} -D_{c}\nabla c. \end{aligned}$$

The parameter $k_{c}$ represents the maximum net secretion rate of the signaling molecule, $\eta ^{I}$ is the ratio of myofibroblasts to fibroblasts in the maximum net secretion rate of the signaling molecule, $a_{c}^{II}$ is the concentration of the signaling molecule that causes the secretion rate of the signaling molecule to be half of its maximum, and $\delta _{c}$ is the proteolytic breakdown rate of the signaling molecules. The parameter $D_{c}$ represents the random diffusion coefficient of the generic signaling molecule. An example of a signaling molecule that can stimulate processes such as the up-regulation of the secretion of collagen molecules by (myo)fibroblasts and the cell differentiation of fibroblasts into myofibroblasts, is transforming growth factor-$\beta$ (TGF-$\beta$) (Barrientos et al. 2008).

The second term on the right hand side of Eq.(10) requires a more detailed introduction. In this study, we incorporate into the model the proteolytic cleavage of the generic signaling molecule by metalloproteinases (MMPs) (Mast and Schultz 1996; Lint and Libert 2007). MMPs are secreted by (myo)fibroblasts and are involved in the breakdown of collagen-rich fibrils during the maintenance and the remodeling of the extracellular matrix (ECM) (Chakraborti et al. 2003; Lindner et al. 2012; Nagase et al. 2006). The secretion of the MMPs is inhibited by the presence of signaling molecules such as TGF-$\beta$ (Overall et al. 1991). Therefore, we assume that the concentration of the MMPs is a function of the cell density of the (myo)fibroblasts, the concentration of the collagen molecules, and the concentration of the signaling molecules:

12 $$\begin{aligned} g(N,M,c,\rho ) = \frac{\left[ N + \eta ^{II}M\right] \rho }{1 + a_{c}^{III}c}. \end{aligned}$$

The parameter $\eta ^{II}$ is the ratio of myofibroblasts to fibroblasts in the secretion rate of the MMPs, and $1/[1 + a_{c}^{III}c]$ represents the inhibition of the secretion of the MMPs due to the presence of the signaling molecule.

### The collagen molecules

The functional forms for the biochemical kinetics associated with the collagen molecules and the functional form for the transportation of these molecules are basically identical to functional forms used previously (Koppenol et al. 2017b):

13 $$\begin{aligned}&\mathbf {J}_{\rho } = \mathbf {0}, \end{aligned}$$

14 $$\begin{aligned}&R_{\rho } = k_{\rho }\left\{ 1 + \left[ \frac{k_{\rho }^{\max }c}{a_{c}^{IV} + c}\right] \right\} \left[ N +\,\eta ^{I} M\right] \nonumber \\&\quad -\,\delta _{\rho }g(N,M,c,\rho )\rho . \end{aligned}$$

The parameter $k_{\rho }$ is the collagen molecule secretion rate, $k_{\rho }^{\max }$ is the maximum factor with which the secretion rate can be enhanced due to the presence of the signaling molecule, $a_{c}^{IV}$ is the concentration of the signaling molecule that causes the half-maximum enhancement of the secretion rate, and $\delta _{\rho }$ is the proteolytic breakdown rate of the collagen molecules.

### The mechanical component

In this study, we use the following visco-elastic constitutive relation for the mathematical description of the relationship between the Cauchy stress tensor on the one hand, and the effective strains and displacement velocity gradients on the other hand:

15 $$\begin{aligned}&\mathbf {\sigma } = \mu _{1}\text {sym}(\mathbf {L}) + \mu _{2}\left[ \text {tr}\left( \text {sym}(\mathbf {L})\right) \mathbf {I}\right] \nonumber \\&\quad ~~+\left[ \frac{E({\rho })}{1 + \nu }\right] \left\{ \mathbf {\varepsilon } + \text {tr}(\mathbf {\varepsilon })\left[ \frac{\nu }{1 - 2\nu }\right] \mathbf {I}\right\} , \end{aligned}$$

16 $$\begin{aligned}&E(\rho ) = E^{I}\sqrt{\rho }. \end{aligned}$$

Here $\mu _{1}$ is the shear viscosity, $\mu _{2}$ is the bulk viscosity, $\nu$ is Poisson’s ratio, $E(\rho )$ is the Young’s modulus, and $\mathbf {I}$ is the second-order identity tensor. Like Ramtani et al. (2004; 2002), we assume that the Young’s modulus is dependent on the concentration of the collagen molecules. The parameter $E^{I}$ is a fixed constant.

Furthermore, we incorporate into the model the generation of an isotropic stress by the myofibroblasts due to their pulling on the ECM. This pulling stress is proportional to the product of the cell density of the myofibroblasts and a simple function of the concentration of the collagen molecules (Olsen et al. 1995; Koppenol et al. 2017a, b):

17 $$\begin{aligned}&\mathbf {f} = \nabla \cdot \mathbf {\psi }, \end{aligned}$$

18 $$\begin{aligned}&\mathbf {\psi } = \xi M\left[ \frac{\rho }{R^2 + \rho ^{2}}\right] \mathbf {I}. \end{aligned}$$

The parameter $\mathbf {\psi }$ represents the total generated stress by the myofibroblast population, $\xi$ is the generated stress per unit cell density and the inverse of the unit collagen molecule concentration, and *R* is a fixed constant.

Finally, we assume that the rate of active change of the effective strain is proportional to the product of the amount of effective strain (as suggested by Hall (2009)), the local concentration of the MMPs, the local concentration of the signaling molecule, and the inverse of the local concentration of the collagen molecules. The directions in which the effective strain changes, are determined by both the signs of the eigenvalues related to the effective strain tensor, and the directions of the associated eigenvectors. Taken together, we obtain the following symmetric tensor:

19 $$\begin{aligned} \mathbf {G} = \zeta \left[ \frac{g(N,M,c,\rho )c}{\rho }\right] \mathbf {\varepsilon } = \zeta \left\{ \frac{\left[ N + \eta ^{II}M\right] c}{1 + a_{c}^{III}c}\right\} \mathbf {\varepsilon }, \end{aligned}$$

where $\zeta$ is the rate of morphoelastic change (i.e., the rate at which the effective strain changes actively over time).

### The domain of computation

We assume $u = 0, \partial v/\partial x = \partial w/\partial x = 0, v_{1} = 0, \partial v_{2}/\partial x = \partial v_{3}/\partial x = 0, \varepsilon _{11} = \varepsilon _{12} = \varepsilon _{21} = \varepsilon _{13} = \varepsilon _{31} = 0$, and $\partial \varepsilon _{22}/\partial x = \partial \varepsilon _{33}/\partial x = 0$ hold within the modeled portion of dermal layer for all time *t*, with the *yz*-plane running parallel to the surface of the skin and

20 $$\begin{aligned} \mathbf {u} = \begin{bmatrix} u \\ v \\ w \end{bmatrix},\quad \mathbf {v} = \begin{bmatrix} v_{1} \\ v_{2} \\ v_{3} \end{bmatrix},\quad \text {and}\quad \mathbf {\varepsilon } = \begin{bmatrix} \varepsilon _{11}&\varepsilon _{12}&\varepsilon _{13} \\ \varepsilon _{21}&\varepsilon _{22}&\varepsilon _{23} \\ \varepsilon _{31}&\varepsilon _{32}&\varepsilon _{33} \end{bmatrix}. \end{aligned}$$

Furthermore, we assume that the derivatives of the cell densities and the concentrations of the modeled constituents of the dermal layer are equal to zero in the direction perpendicular to the surface of the skin. Taken together, these assumptions imply that the calculations can be performed on an arbitrary, infinitely thin slice of dermal layer oriented parallel to the surface of the skin, and that the results from these calculations are valid for every infinitely thin slice of dermal layer oriented parallel to the surface of the skin. Therefore, we use the following domain of computation:

21 $$\begin{aligned} \Omega _{\mathbf {X}} \in \{X = 0, -10 \le Y \le 10, -10 \le Z \le 10\}, \end{aligned}$$

where $\mathbf {X} = (X,Y,Z)^{\text {T}}$ are Lagrangian coordinates.

### The initial conditions and the boundary conditions

The initial conditions give a description of the cell densities and the concentrations immediately after placement of the skin graft on its recipient bed. For the generation of the simulation results, the following function has been used to describe the shape of the skin graft:

22 $$\begin{aligned}&w(\mathbf {X}_{r}) = 1 - \left[ 1 - I\left( Y_{r},2.5,0.10\right) \right] \left[ 1 - I\left( Z_{r},2.5,0.10\right) \right] \nonumber \\&\quad \times \, I\left( Y_{r},2.5,0.10\right) I\left( Z_{r},2.5,0.10\right) , \end{aligned}$$

where

23 $$\begin{aligned} I\left( r,s_{1},s_{2}\right) = {\left\{ \begin{array}{ll} 0 &{} \text {if } r < \left[ s_{1} - s_{2}\right] , \\ \frac{1}{2}\left[ 1 + \sin \left( \frac{\left[ r - s_{1}\right] \pi }{2s_{2}}\right) \right] &{} \text {if } \left| r - s_{1}\right| \le s_{2}, \\ 1 &{} \text {if } r > \left[ s_{1} + s_{2}\right] . \end{array}\right. }\nonumber \\ \end{aligned}$$

Here $w = 0$ corresponds to grafted dermis and $w = 1$ corresponds to unwounded dermis. The values for the parameters $s_{1}$ and $s_{2}$ determine, respectively, the location of the boundary between the skin graft and the undamaged dermis, and the minimum distance between completely grafted dermis and unwounded dermis. Furthermore, $\mathbf {X}_{r} = \mathbf {R}(\theta _{r})\mathbf {X} = (X_{r},Y_{r},Z_{r})^{\text {T}}$ with $\mathbf {R}(\theta )$ the counterclockwise rotation matrix that rotates vectors by an angle $\theta$ about the *X*-axis, and $\theta _{r} = \pi /4\ \text {rad}$.

**Fig. 1:**
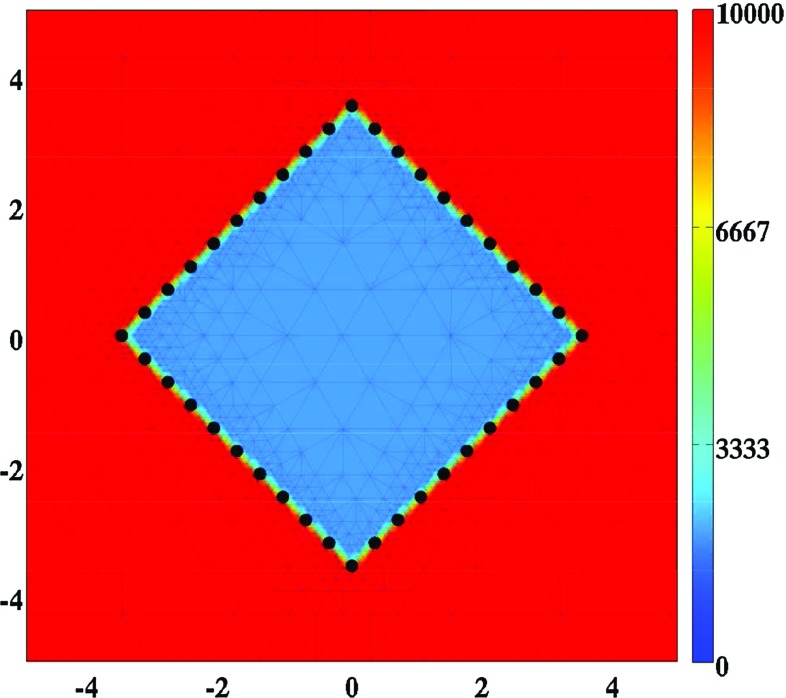
A graphical overview of the initial conditions. Depicted are the initial shape of the skin graft and, in color scale, the initial cell density of the fibroblasts ($\text {cells}/\text {cm}^{3}$). The scale along both axes is in centimeters. The *X*-axis points toward the reader. The *black dots* mark the material points that were used to trace the evolution of the surface area of the skin graft over time. That is, at each time point, the area of the polygon with vertices located at the displaced black material points has been determined

Based on the function for the shape of the skin graft, we take the following initial conditions for the modeled constituents of the dermal layer:

24 $$\begin{aligned}&N(\mathbf {X},0) = \left\{ I^{w} + \left[ 1 - I^{w}\right] w(\mathbf {X}_{r})\right\} \overline{N}, \nonumber \\&M(\mathbf {X},0) = \overline{M}, \nonumber \\&c(\mathbf {X},0) = [1 -w(\mathbf {X}_{r})]c^{w},\nonumber \\&\rho (\mathbf {X},0) = \overline{\rho }. \end{aligned}$$

Here $\overline{N}, \overline{M}$, and $\overline{\rho }$ are, respectively, the equilibrium cell density of the fibroblasts, the equilibrium cell density of the myofibroblasts, and the equilibrium concentration of the collagen molecules, of the unwounded dermis. Due to the secretion of signaling molecules by for instance leukocytes, signaling molecules are present in the wounded area. The constant $c^{w}$ represents the maximum initial concentration of the signaling molecule in the grafted area. Furthermore, we assume that there are some fibroblasts present in the grafted area. The value for the parameter $I^{w}$ determines how much fibroblasts are present minimally initially in the grafted area.

With respect to the initial conditions for the mechanical component of the model, we take the following initial conditions for all $\mathbf {x} \in \Omega _{\mathbf {x},0}$ where $\Omega _{\mathbf {x},0}$ is the initial domain of computation in Eulerian coordinates:

25 $$\begin{aligned} \mathbf {u}(\mathbf {x},0) = \mathbf {0},\quad \mathbf {v}(\mathbf {x},0) = \mathbf {0},\quad \text {and}\quad \mathbf {\varepsilon }(\mathbf {x},0) = \mathbf {0}. \end{aligned}$$

See Fig. 1 for a graphical representation of the initial conditions that have been used in this study.

With respect to the boundary conditions for the constituents of the dermal layer, we take the following Dirichlet boundary conditions for all time *t* and for all $\mathbf {x} \in \partial \Omega _{\mathbf {x},t}$ where $\partial \Omega _{\mathbf {x},t}$ is the boundary of the domain of computation in Eulerian coordinates:

26 $$\begin{aligned} N(\mathbf {x},t) = \overline{N},\quad M(\mathbf {x},t) = \overline{M},\quad \text {and}\quad c(\mathbf {x},t) = \overline{c}. \end{aligned}$$

The parameter $\overline{c}$ is the equilibrium concentration of the signaling molecule in the unwounded dermis.

Finally, with respect to the boundary condition for the mechanical component of the model, we take the following Dirichlet boundary condition for all time *t* and for all $\mathbf {x} \in \partial \Omega _{\mathbf {x},t}$:

27 $$\begin{aligned} \mathbf {v}(\mathbf {x},t) = \mathbf {0}. \end{aligned}$$

**Fig. 2:**
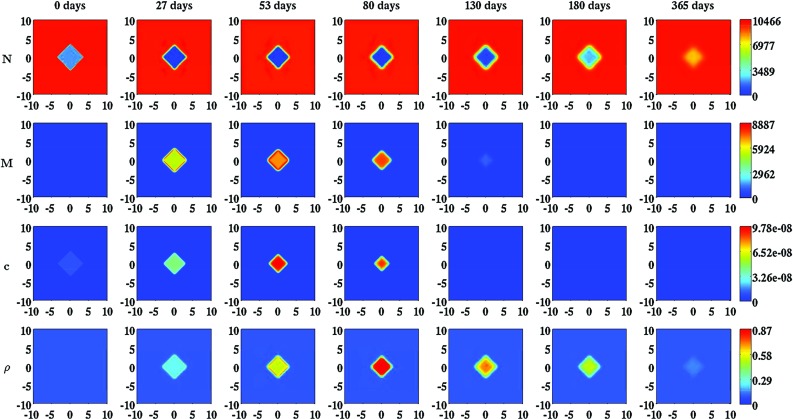
An overview of simulation results for the modeled constituents of the dermal layer when the inhibition of the secretion of MMPs due to the presence of signaling molecules is relatively low ($a_{c}^{III} = 2\times 10^{8}\ \text {cm}^{3}/\text {g}$) and the rate of morphoelastic change is relatively high ($\zeta = 9\times 10^{2}\ \text {cm}^{6}/(\text {cells g day})$). The values for all other parameters are equal to those depicted in Table 1 in Appendix 3. The *top two rows* show the evolution over time of the cell density of, respectively, the fibroblast population and the myofibroblast population. The *color scales* represent the cell densities, measured in $\text {cells}/\text {cm}^{3}$. The *bottom two rows* show the evolution over time of the concentrations of, respectively, the signaling molecules and the collagen molecules. The *color scales* represent the concentrations, measured in $\text {g}/\text {cm}^{3}$. Within the subfigures, the scale along both axes is in centimeters

### The parameter value estimates

Table 1 in Appendix 3 provides an overview of the dimensional (ranges of the) values for the parameters of the model. The majority of these values were either obtained directly from previously conducted studies or estimated from results of previously conducted studies. In addition, we were able to determine the values for three more parameters due to the fact that these values are a necessary consequence of the values chosen for the other parameters (Koppenol et al. 2017b).

## Simulation results

**Fig. 3:**
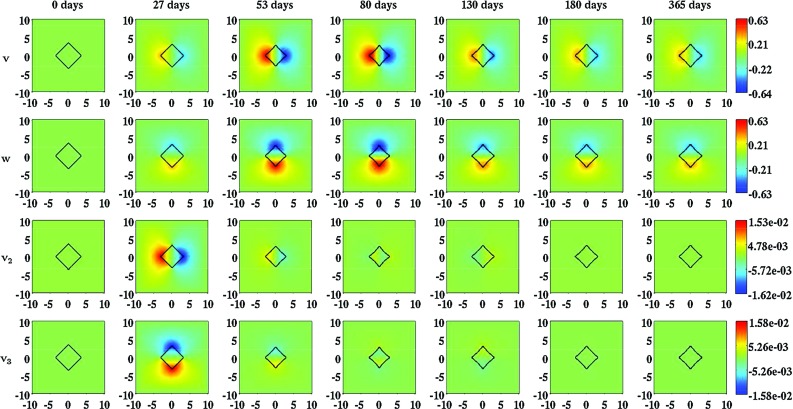
An overview of simulation results for the displacement field and the displacement velocity field when the inhibition of the secretion of MMPs due to the presence of signaling molecules is relatively low ($a_{c}^{III} = 2\times 10^{8}\ \text {cm}^{3}/\text {g}$) and the rate of morphoelastic change is relatively high ($\zeta = 9\times 10^{2}\ \text {cm}^{6}/(\text {cells g day})$). The values for all other parameters are equal to those depicted in Table 1 in Appendix 3. The *top two rows* show the evolution over time of the displacement in, respectively, the horizontal direction and the vertical direction. The *color scales* represent the displacements, measured in centimeters. The *bottom two rows* show the evolution over time of the displacement velocity in, respectively, the horizontal direction and the vertical direction. The *color scales* represent the displacement velocities, measured in $\text {cm}/\text {day}$. Within the subfigures, the scale along both axes is in centimeters. The *black squares* within the subfigures represent the (displaced) boundaries between the skin graft and the unwounded dermis

**Fig. 4:**
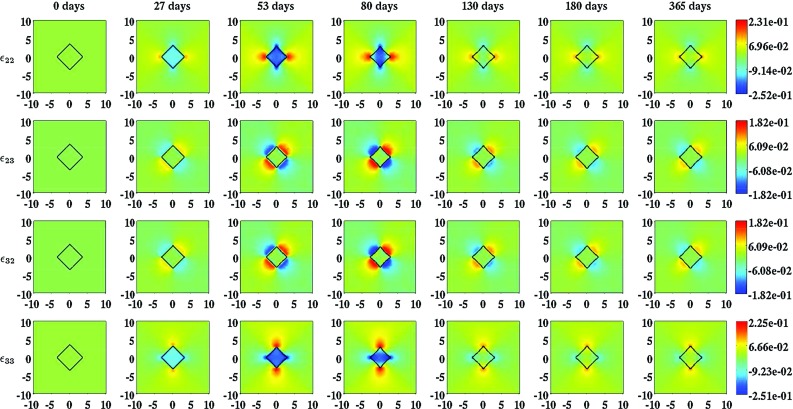
An overview of simulation results for the effective strain when the inhibition of the secretion of MMPs due to the presence of signaling molecules is relatively low ($a_{c}^{III} = 2\times 10^{8}\ \text {cm}^{3}/\text {g}$) and the rate of morphoelastic change is relatively high ($\zeta = 9\times 10^{2}\ \text {cm}^{6}/(\text {cells g day})$). The values for all other parameters are equal to those depicted in Table 1 in Appendix 3. The *separate rows* show the evolution over time of the different components of the effective strain that are unequal to zero. The *color scales* represent the amount of effective strain. Within the subfigures, the scale along both axes is in centimeters. The *black squares* within the subfigures represent the (displaced) boundaries between the skin graft and the unwounded dermis

In order to obtain some insight into the dynamics of the model, we present an overview of simulation results for the modeled constituents of the dermal layer in Fig. 2. Furthermore, we present an overview of simulation results for the displacement field and the displacement velocity field in Fig. 3, and an overview of simulation results for the effective strain in Fig. 4. For the generation of these overviews, the same set of values for the parameters of the model was used.

**Fig. 5:**
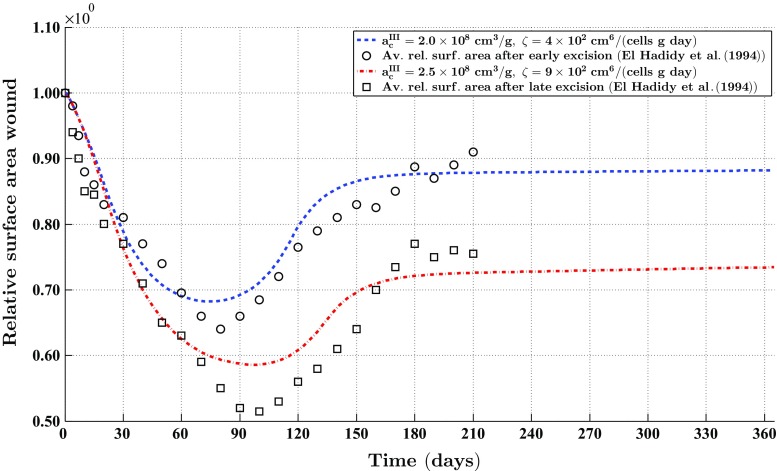
The evolution over time of the relative surface area of wounds (i.e., skin grafts) for particular combinations of values for the rate of morphoelastic change (i.e., the parameter $\zeta$), and the parameter related to the inhibition of the secretion of MMPs due to the presence of signaling molecules (i.e., the parameter $a_{c}^{III}$). The values for all other parameters are equal to those depicted in Table 1 in Appendix 3. The *black circles* and the *black squares* show the evolution over time of the average of clinical measurements of the relative surface areas of placed unmeshed skin grafts after, respectively, early excision of burnt skin and late excision of burnt skin (El Hadidy et al. 1994)

Figure 2 shows that the cell density of the myofibroblasts, and the concentrations of both the signaling molecules and the collagen molecules increase first within the skin graft. Subsequently, the concentrations of these molecules, just like the cell density of the myofibroblasts, start to decline until they reach the equilibrium concentrations and the equilibrium cell density of uninjured dermal tissue. Meanwhile, the cell density of the fibroblasts starts to increase within the skin graft until it reaches the equilibrium cell density of uninjured dermal tissue.

**Fig. 6:**
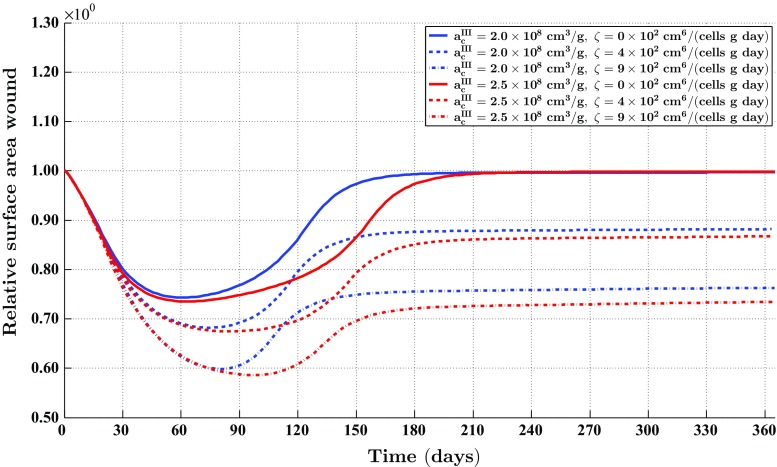
The evolution over time of the relative surface area of wounds (i.e., skin grafts) for some combinations of values for the rate of morphoelastic change (i.e., the parameter $\zeta$), and the parameter related to the inhibition of the secretion of MMPs due to the presence of signaling molecules (i.e., the parameter $a_{c}^{III}$). The values for all other parameters are equal to those depicted in Table 1 in Appendix 3

Figure 3 shows that the boundaries between the skin graft and the uninjured tissue are pulled inward increasingly toward the center of the skin graft while the concentration of the collagen molecules and the cell density of the myofibroblasts increase. Looking at the displacement velocity field, we observe that the boundaries are pulled inward relatively fast initially. Subsequently, the speed with which the boundaries are pulled inward diminishes fast. Looking carefully at the displacement velocity field, we observe that the inward movement actually reverses from a certain time point onward. It is nice to observe that this phenomenon coincides with the gradual increase in the surface area of the skin graft, and the gradual decrease in both the cell density of the myofibroblasts and the concentration of the collagen molecules within the skin graft, as can be observed in, respectively, Fig. 6 and Fig 2. Furthermore, we observe that the boundaries between the skin graft and the uninjured tissue hardly move anymore eventually (i.e., the individual components of the displacement velocity field become approximately equal to zero over the domain of computation), and that the surface area of the skin graft has diminished considerably after a year. This latter phenomenon is also clearly visible in Fig. 6.

Figure 4 also shows something very interesting. If we look at the effective strain at day 365, we observe that the individual components of the effective strain tensor are not equal to zero over the domain of computation. This implies that there are residual stresses present in the grafted area. Comparing the properties of the effective strain at day 180 with the properties of the effective strain at day 365, we observe that these are more or less the same. Hence, the residual stresses remain present in the modeled portion of dermal layer for a prolonged period of time.

Figure 5 shows the evolution over time of the relative surface area of skin grafts for particular combinations of values for two parameters that are directly related to the tensor $\mathbf {G}$ (See Eq. (19)). In addition, the figure shows averages of clinical measurements over time of the relative surface areas of placed unmeshed skin grafts in human subjects after both early excision of burnt skin and late excision of burnt skin (El Hadidy et al. 1994).

Furthermore, Fig. 6 shows the evolution over time of the relative surface area of skin grafts for some more combinations of values for the aforementioned parameters related to the tensor $\mathbf {G}$. The figure shows that both an increase in the rate of morphoelastic change (i.e., the parameter $\zeta$), and an increase in the inhibition of the secretion of MMPs due to the presence of signaling molecules (i.e., an increase in the value for the parameter $a_{c}^{III}$) results in a reduction of the final surface area of a skin graft. Within the chosen ranges for the values of the parameters, we observe that a change in the value for the rate of morphoelastic change has a large impact on the final surface area of a skin graft. Changing the value for the parameter related to the inhibition of the secretion of MMPs due to the presence of signaling molecules has a smaller impact on the final surface area of a skin graft. Note also that the value for the latter parameter has a relatively large impact on the total number of days that the boundaries between the skin graft and the uninjured tissue are pulled inward after placement of the skin graft before the retraction process starts.

Finally, it is nice to observe in Fig. 6 that, as expected, the surface area of a skin graft returns to its initial value when the rate of morphoelastic change is equal to zero. If this rate is equal to zero, then the tensor $\mathbf {G}$ is equal to the zero tensor. In this case, one would expect an initial period during which the surface area of a skin graft diminishes due to the pulling action of the myofibroblasts, followed by a period during which this surface area slowly returns to its initial value due to the apoptosis of the myofibroblasts. This is exactly what can be observed in the figure.

## Discussion

We have presented a continuum hypothesis-based model for the simulation of the (long term) contraction of skin grafts that cover excised burns. Since skin contraction and contracture formation mainly take place in the dermal layer of the skin, we incorporated solely a portion of this layer into the model. The dermal layer is modeled as a heterogeneous, isotropic, morphoelastic solid with a Young’s modulus that is locally dependent on the concentration of the collagen molecules. For this end, we used the theory of morphoelasticity developed by Hall (2009). In particular, we used in this study the derived evolution equation that describes the dynamic change of the infinitesimal effective strain over time. Furthermore, we used the general conservation equations for linear momentum and mass to describe mathematically the dynamic change over time of, respectively, the linear momentum, and the cell densities and concentrations of the modeled constituents of the dermal layer. For the description of the relationship between the Cauchy stress tensor on the one hand, and the effective strain tensor and displacement velocity gradients on the other hand, we used the visco-elastic constitutive relation given in Eq. (15).

Related to the mechanical component of the model, we want to remark the following. Traditionally, the dermis is modeled as a linear visco(elastic) solid in mechano-chemical continuum models for dermal wound healing (Javierre et al. 2009; Murphy et al. 2012; Olsen et al. 1995; Ramtani 2004; Ramtani et al. 2002; Valero et al. 2014a, b; Vermolen and Javierre 2012). More recently, continuum models have appeared where the dermis is modeled as a hyperelastic solid (Koppenol et al. 2017a; Valero et al. 2013, 2015). Unfortunately, it is difficult with any of these models to simulate the long term deformation of dermal tissues and the development of residual stresses within these tissues while these phenomena are often observed in the medical clinic (Schouten et al. 2012). Therefore, we adopted like Murphy et al. (2011) and Bowden et al. (2016), a morphoelastic framework in this study. With the application of such a framework, it becomes relatively simple to simulate both the long term deformation of a skin graft and the development of residual stresses within the modeled portion of dermal layer.

With respect to the constituents of a recovering injured area, we selected the following four constituents as primary model variables: fibroblasts, myofibroblasts, a generic signaling molecule, and collagen molecules. The mathematical descriptions for the movement of the cells, the biochemical kinetics associated with these cells, the dispersion of the generic signaling molecule, and the release, consumption, and removal of both the collagen molecules and the generic signaling molecule are nearly identical to the functional forms used previously (Koppenol et al. 2017b).

Furthermore, we present an overview of the applied numerical algorithm that has been developed for the generation of computer simulations in Appendix 1. The development of this algorithm was necessary to “catch” the local dynamics of the model and obtain sufficiently accurate simulations within an acceptable amount of CPU time. For this end, we combined a moving-grid finite-element method (Madzvamuse et al. 2003) with an element resolution refinement / recoarsement method (Möller et al. 2008) and an automatically adaptive time-stepping method (Kavetski et al. 2002). We present the derivation of the general finite-element approximation in Appendix 2. Furthermore, we applied both a source term splitting procedure (Patankar 1980) and a semi-implicit flux-corrected transport (FCT) limiter (Möller et al. 2008) on the discretized system of equations that describes the dynamics of the modeled constituents of the dermal layer in order to guarantee the positivity of the approximations of the solutions for these primary model variables.

With the developed model, it is possible to simulate some general qualitative features of the healing response that is initiated after the placement of a skin graft on its recipient bed (Harrison and MacNeil 2008). The restoration of the presence of fibroblasts within the skin graft and the temporary presence of myofibroblasts during the execution of the healing response can be simulated. Due to the initial presence of signaling molecules and the gradual increase in the cell density of the myofibroblasts in the grafted area, the secretion rate of collagen molecules is considerably larger than the proteolytic breakdown rate of these molecules in the grafted area for a prolonged period of time (See also Eq. (14)). Consequently, the concentration of the collagen molecules in the grafted area becomes substantially higher than the concentration of the collagen molecules in the surrounding uninjured dermal tissue before it gradually decreases toward the concentration of the collagen molecules in the surrounding uninjured dermal tissue. Furthermore, it is possible to simulate both the long term contraction and subsequent retraction of a skin graft, and the development of residual stresses within the dermal layer. These phenomena can be observed, respectively, in Figs. 3 and 4; both the displayed components of the displacement field and the displayed components of the effective strain tensor are not equal zero over the domain of computation at day 365, and the values of the individual components over the domain of computation at day 365 are roughly equal to the values of the individual components over the domain of computation at day 180. Looking at the individual components of the displacement velocity field in Fig. 3, it can be observed that these have become approximately equal to zero over the domain of computation at day 365.

Focusing on the simulation of the contraction of skin grafts and the formation of contractures we observe the following. Figure 5 shows a good match with respect to the evolution over time of the relative surface area of skin grafts between measurements obtained in a clinical study by El Hadidy et al. (1994) and outcomes of computer simulations obtained in this study. This agreement provides us some confidence about the validity of the model. Obviously, the number of models with which it is possible to produce the depicted contraction curves is infinite in theory. Therefore, we would have liked to validate the presented model against scar assessment data of a different kind such as cell density profiles and collagen molecule concentration profiles, in order to increase our confidence about the validity of the model. However, we have not been able to find more appropriate experimental measurement data in the available literature. We are not the only ones who have to deal with this issue. Unfortunately, it is a fundamental problem in the field of mathematical modeling of dermal wound healing processes to find suitable experimental measurement data for the proper validation of models (Bowden et al. 2016). In our opinion, this does not imply that we should refrain from deducing biomedical implications from the results obtained in this study. However, we do think that it is very important to be careful when doing so, and to keep in mind that these deductions are based on outcomes of a mathematical modeling study.

Having said that, we focus now on the implications of the results depicted in Fig. 5. In this study, we assumed that the rate at which the effective strain is changing actively over time is proportional to the product of the amount of effective strain, the local concentration of the MMPs, the local concentration of the signaling molecule, and the inverse of the local concentration of the collagen molecules. The directions in which the effective strain changes, are determined by both the signs of the eigenvalues related to the effective strain tensor, and the directions of the associated eigenvectors. The good match between the gathered scar assessment data and the outcomes of the computer simulations suggests that this combination of relationships might describe appropriately in mathematical terms the mechanism underlying the formation of contractures.

If the mathematical description for the mechanism underlying the formation of contractures is indeed appropriate, then this suggests the following. Looking at Eq. (19), it is clear that the effective strain can change solely when the local concentration of the signaling molecules is unequal to zero. Given the presence of signaling molecules within the grafted area immediately after placement of the skin graft on its recipient bed, this implies that the optimal point in time to start with splinting therapy is directly after surgery. It is interesting to note that this implication matches nicely with the finding that early mechanical restraint of tissue-engineered skin leads to a reduction in the extent of contraction (Harrison and MacNeil 2008). Furthermore, it is also evident that it is desirable to continue with splinting therapy until the concentration of the signaling molecules in the grafted area has become negligible such that the formation of contractures can be prevented. Given that it is unclear at present what the optimal point in time is after surgery to start with splinting therapy, and how long the static splints have to be worn for the therapy to be effective (Schouten et al. 2012), these are interesting observations.

Furthermore, Fig. 5 shows that the difference in the evolution over time of the average of the relative surface areas of placed unmeshed skin grafts between grafts that are placed after early excision of the burnt skin and grafts that are placed after late excision of the burnt skin might be caused by both a change in the rate of morphoelastic change and a change in the degree of inhibition of the secretion of MMPs due to the presence of signaling molecules. In itself, this is an interesting observation. In addition, it provides us with some alternative ideas on how to diminish the degree of contracture formation that are not based on a mechanical intervention. As demonstrated in Fig. 6, the final surface area of a skin graft can be increased by both a reduction in the rate of morphoelastic change, and a reduction in the inhibition of the secretion of MMPs due to the presence of signaling molecules. Within the investigated ranges of values, the former reduction has a huge impact on the final surface area whereas the latter reduction has a smaller impact on the final surface area. Perhaps that the reduction in the rate of morphoelastic change can be accomplished through the local inhibition of certain cross-linking enzymes. For instance, perhaps it is possible to use the chemical $\beta$-aminopropionitrile (BAPN) for the inhibition of the cross-linking enzyme lysyl oxidase, which is crucial for the stabilization of collagen fibrils (Kagan and Li 2003; Wilmarth and Froines 1992). The reduction in the inhibition of the secretion of MMPs due to the presence of signaling molecules can be accomplished perhaps by influencing the regulation of the transcription of MMPs by signaling molecules such as TGF-$\beta$ (Overall et al. 1991). Given that it is actually unclear at present how effective splinting therapy is in preventing contracture formation (Schouten et al. 2012), we think that these suggestions, which are not based on a mechanical intervention, could be interesting alternatives for the reduction of the degree of contracture formation.

We want to conclude this section with a short discussion about how the presented model can be adjusted. We want to mention here a couple of ways in which the model can be adjusted. In this study, we made some assumptions which made it possible to perform the calculations on an infinitely thin slice of dermal layer oriented parallel to the surface of the skin. An obvious benefit of this is that it results in a serious reduction of the computation times to obtain computer simulations. However, it is probably very interesting to investigate in a three-dimensional portion of dermal layer what would happen to the (long term) contraction and subsequent retraction of skin grafts. For this end, the main adaptations to the model presented in this study would be the removal of the assumptions made in Sect. 2.5, and the introduction of additional boundary conditions for the interfaces between the epidermis and the dermis and the subcutaneous tissue and the dermis. In a three-dimensional setting, we could use, for example, the spring-like boundary conditions introduced in the study by Koppenol et al. (2017b) to describe the attachment of the dermal tissues to the subcutaneous tissue.

Unfortunately, there is no information presented about the shapes and the absolute surface areas of the excised burns in the study by El Hadidy et al. (1994). Therefore, we decided to use the same shape for the grafted area in all computer simulations. Obviously, it is easy to obtain computer simulations with the presented model for grafted areas with a different shape and / or surface area. The only element of the model that needs to be adjusted is the function given in Eq. (22). In particular, when more detailed clinical measurement data about the evolution over time of the shapes of skin grafts become available in the scientific literature, it will become very interesting to investigate to what extent the presented model in this study can replicate such clinical measurement data. Furthermore, the presented model could also be used for the simulation of the healing of dermal wounds that heal by secondary intention alone (i.e., without the placement of a skin graft). The only element of the model that would really need to be adjusted is the initial condition for the collagen molecules presented in Eq. (24).

In this study we assumed that the effective strains are small. As a consequence, we used in this study the evolution equation that describes the dynamic change of the infinitesimal effective strain over time (i.e., Eq. (1c)). However, it might actually be more appropriate to assume that the effective strains can become arbitrary large. If we make this assumption, then we can replace Eq. (1c) with the following evolution equation that gives a description of the dynamic change of the Eulerian finite effective strain ($\mathbf {e}$) over time (Hall 2009):

28 $$\begin{aligned} \frac{\text {D}\mathbf {e}}{\text {D}t} = \text {sym}\left( \mathbf {B}^{-1}\mathbf {L} - \frac{1}{\sqrt{\text {det}\left( \mathbf {B}^{-1}\right) }}\mathbf {B}^{-1}\mathbf {G}\right) , \end{aligned}$$

where

29 $$\begin{aligned} \mathbf {e} = \frac{1}{2}\left\{ \mathbf {I} - \left[ \mathbf {A}^{-1}\right] ^{2}\right\} , \end{aligned}$$

and

30 $$\begin{aligned} \mathbf {B} = \left[ \mathbf {I} - 2\mathbf {e}\right] ^{-1}. \end{aligned}$$

Subsequently, we can replace the constitutive relation presented in Eq. (15) with, for instance, the following constitutive relation:

31 $$\begin{aligned} \mathbf {\sigma }= & {} \mu _{1}\text {sym}(\mathbf {L}) + \mu _{2}\left[ \text {tr}\left( \text {sym}(\mathbf {L})\right) \mathbf {I}\right] \nonumber \\&+ \left\{ \left\{ \frac{E(\rho )}{2\left[ 1 - 2\nu \right] }\right\} \left[ \sqrt{\text {det}\left( \mathbf {B}\right) } - 1\right] \right\} \mathbf {I}\nonumber \\&+\left\{ \frac{E(\rho )}{2\left[ 1 +\nu \right] }\right\} \left[ \sqrt{\text {det}\left( \mathbf {B}\right) }\right] ^{-\frac{5}{3}}\left[ \mathbf {B} - \frac{1}{3}\text {tr}\left( \mathbf {B}\right) \mathbf {I}\right] . \end{aligned}$$

Here the elastic component of the constitutive relation is equal to constitutive relation for a heterogeneous, isotropic and compressible neo-Hookean solid (Koppenol et al. 2017b; Treloar 1948).

The second way in which the mechanical component of the model can be adjusted is the following. It is known that the way that collagen molecules are organized into interconnected sheets and bundles influences the response of dermal tissues to mechanical forces (Jor et al. 2011). Therefore, we have developed a continuum hypothesis-based model in which the bulk mechanical behavior of the involved dermal tissues is dependent on the geometrical arrangement of the collagen bundles (Koppenol et al. 2017a). In this model, a tensorial approach is used to represent the collagen bundles, and the bulk mechanical properties of the tissues such as the Young’s moduli and Poisson ratios are dependent on both the local concentration and the local geometrical arrangement of these collagen bundles. Hence, the model we have presented here can be adjusted by using the tensorial approach for the representation of collagen bundles, and by replacing the elastic component of the constitutive relation given in Eq. (15) with the constitutive relation derived in the study by Koppenol et al. (2017a). Due to these adaptations to the model presented in this study, it becomes possible to study the impact of tissue anisotropy on the deformation of skin grafts during healing.

## Appendix 1: The applied numerical algorithm

In this section, we present an overview of the numerical algorithm that we developed for the generation of computer simulations. The development of this algorithm was necessary to “catch” the local dynamics of the model, obtain sufficiently accurate simulations within an acceptable amount of CPU time, and guarantee the positivity of the approximations of the solutions for the modeled constituents of the dermal layer.

For the kernel of the concrete expression of the algorithm we used MATLAB together with its Parallel Computing Toolbox (MathWorks 2014). Furthermore, we interfaced this kernel consecutively with an adapted version of the mesh generator developed by Persson and Strang (2004) for the generation of a base triangulation of the domain of computation, the element resolution refinement / recoarsement tool of the computational fluid dynamics software package FEATFLOW2 for the adjustment of the resolution of the elements of the base triangulation (Turek 1998), and the permutation routine HSL_MC64 for the permutation of the $n \times n$ matrices related to the resulting systems of linear algebraic equations after full discretization of the model equations such that the matrices have *n* entries on their diagonal (HSL 2013). Finally, we applied the following nondimensionalisation to the model:

32 $$\begin{aligned} \begin{aligned} x&= Lx^{*},&t&= \left[ L^{2}/\left[ D_{F}\overline{N}\right] \right] t^{*},&\rho&= \overline{\rho }\rho ^{*},&N&= \overline{N}N^{*}, \\ \mathbf {u}&= L\mathbf {u}^{*},&\mathbf {v}&= \left[ \left[ D_{F}\overline{N}\right] /L\right] \mathbf {v}^{*},&c&= c^{w}c^{*},&M&= \overline{N}M^{*},\\ \mathbf {\varepsilon }&= \mathbf {\varepsilon }^{*},&\mathbf {\sigma }&= \left[ \left[ \xi \overline{N}\right] /\overline{\rho }\right] \mathbf {\sigma }^{*}, \end{aligned}\nonumber \\ \end{aligned}$$

where $L = 1\ \text {cm}$ is the length scale of the model. The variables with the asterisks are the nondimensionalised variables.

In the following paragraphs, we present a step-by-step description of the algorithm. Basically, the algorithm consists of two parts. The first part of the algorithm is dedicated to the generation of a proper triangulation of the domain of computation. The second part of the algorithm is dedicated to obtaining an approximation of the solution for the primary model variables from Eq. (1c) after application of the nondimensionalisation.

In order to create a conforming base triangulation we used the adapted version of the mesh generator developed by Persson and Strang (2004). This results in a triangulation of the domain of computation where most of the triangles are equilateral and have an average initial edge length of $1.65\ \text {cm}$. The triangles that are not equilateral, are located near the boundary of the domain of computation. Using the following measure for the quality of a triangle *ABC*:

33 $$\begin{aligned} \alpha (ABC) = 2\sqrt{3}\left[ \frac{\Vert CA \times CB\Vert }{\Vert CA\Vert ^{2} + \Vert AB\Vert ^{2} + \Vert BC\Vert ^{2}}\right] , \end{aligned}$$

we observed that $\alpha > 0.86$ for all triangles in the generated triangulation, where $0 \le \alpha \le 1$ and $\alpha = 1$ for equilateral triangles (Lo 1989). Hence, the triangles that are not equilateral are nearly equilateral.

After the generation of the base triangulation, we used the element resolution refinement / recoarsement tool to adjust the resolution of the elements of the base triangulation (Möller 2008). For this purpose, the $L_{2}$-norm of an estimation of the error in the gradient of the numerical approximation of the function that gives a mathematical description of the shape of the skin graft (i.e., Eq. (22)) per element is determined first (Möller and Kuzmin 2006). Subsequently, the resolution of the elements is adapted in order to adjust the estimated error. For this end, we used the following measure to determine the relative error per element $K_{t}$ in the gradient of the numerical approximation of the function that gives a mathematical description of the shape of the skin graft with respect to the other elements:

34 $$\begin{aligned} \beta (K_{t}) =\left[ \frac{\left| \mathcal {T}_{h,t}\right| \left\| \hat{\mathbf {e}}\right\| _{L_{2}(K_{t})}^{2}}{{\sum \limits _{K_{t} \in \mathcal {T}_{h,t}}}\left( \left\| \sigma _{h}\right\| _{L_{2}(K_{t})}^{2} +\left\| \hat{\mathbf {e}}\right\| _{L_{2}(K_{t})}^{2}\right) }\right] ^{\frac{1}{2}}. \end{aligned}$$

Here, $\mathcal {T}_{h,t}$ represents the current triangulation, $\left| \mathcal {T}_{h,t}\right|$ represents the number of elements that constitute this current triangulation, $\left\| \hat{\mathbf {e}}\right\| _{L_{2}(K_{t})}$ represents the $L_{2}$-norm of the estimation of the error in the gradient of the numerical approximation of the function that gives a mathematical description of the shape of the skin graft over element $K_{t}$, and $\left\| \sigma _{h}\right\| _{L_{2}(K_{t})}$ represents the $L_{2}$-norm of a low-order estimation of the gradient of the numerical approximation of the function that gives a mathematical description of the shape of the skin graft over element $K_{t}$ (Möller and Kuzmin 2006). If $\beta (K_{t}) > 0.2$, then the resolution of the element $K_{t}$ is increased. If $\beta (K_{t}) < 0.04$, then the resolution of the element $K_{t}$ is decreased. In this study, the resolution of the elements in the base triangulation can be increased at most four times and the size of the elements in the base triangulation cannot be increased beyond the size they have in this base triangulation.

The estimation of the error $\left\| \hat{\mathbf {e}}\right\| _{L_{2}(K_{t})}$ and the subsequent adjustment of the resolution of the elements are repeated until either the absolute value of the relative change of the sum of the $L_{2}$-norm of the estimation of the error of the gradient over all elements is smaller than 5%, or the maximum number of allowed for iterations is reached. In this study we set this latter number to ten.

As was mentioned before, the second part of the algorithm is dedicated to obtaining an approximation of the solution for the primary model variables from Eq. (1c). In order to find such an approximation, we used the method of lines together with the standard fixed-point defect correction method (Van Kan et al. 2014). The equations from Eq. (1c) are solved in a segregated way. That is, each fixed-point iteration within each time step approximations of the solutions for the modeled constituents of the dermal layer are determined together first, and subsequently approximations of the solutions for the displacement velocity and the effective strain are determined by solving Eq. (1b) and Eq. (1c) simultaneously. Finally, using the fact that the following holds

35 $$\begin{aligned} \mathbf {u}(\mathbf {x}(\mathbf {X},t),t) = \mathbf {U}(\mathbf {X},t),\quad \text {and}\quad \mathbf {v}(\mathbf {x}(\mathbf {X},t),t) = \mathbf {V}(\mathbf {X},t), \end{aligned}$$

where $\mathbf {U}$ and $\mathbf {V}$ are, respectively, the displacement and the displacement velocity of the dermal layer in Lagrangian coordinates, we determine an approximation of the solution for the displacement of the dermal layer from Eq. (2) via postprocessing in the following way:

36 $$\begin{aligned} \mathbf {U}_{n+1}^{(m+1)} = \mathbf {U}_{n} + \Delta t\left\{ \left[ 1 - \theta \right] \mathbf {V}_{n} + \theta \mathbf {V}_{n+1}^{(m+1)}\right\} . \end{aligned}$$

Here, $\mathbf {U}_{n}$ and $\mathbf {V}_{n}$ are, respectively, the final approximations of the solutions for the displacement and the displacement velocity after *n* time steps. Furthermore, $\mathbf {U}_{n+1}^{(m+1)}$ and $\mathbf {V}_{n+1}^{(m+1)}$ are, respectively, the new approximations of the solutions for the displacement and the displacement velocity after application of $m+1$ fixed-point iteration(s). The parameter $\Delta t$ is the size of the current time step and the parameter $\theta$ is a fixed constant which is set to 0.55 in this study. After obtaining a new approximation of the solution for the displacement of the dermal layer, the position of the vertices in the triangulation is updated in the following way:

37 $$\begin{aligned} \mathbf {x}_{j}^{(m+1)}(\mathbf {X}_{j},t) = \mathbf {X}_{j} + \mathbf {U}_{j}^{(m+1)}(\mathbf {X}_{j},t). \end{aligned}$$

Here $\mathbf {x}_{j}^{(m+1)}(\mathbf {X}_{j},t)$ is the new location of vertex *j* in Eulerian coordinates after application of $m+1$ fixed-point iteration(s), $\mathbf {X}_{j}$ is the fixed location of vertex *j* in Lagrangian coordinates, and $\mathbf {U}_{j}^{(m+1)}(\mathbf {X}_{j},t)$ is the new approximation of the solution for the displacement at vertex *j* after application of $m+1$ fixed-point iteration(s).

The fixed-point defect correction scheme is iterated until both the maximum of the relative 1-norms of the residuals of the approximations is smaller than one, and the maximum of the relative 1-norms of the difference between subsequent approximations per variable is smaller than $5\times 10^{-2}$. If the correction scheme does not meet these convergence criteria within five iterations, then the scheme is interrupted, the time step is decreased to 85% of its current value, and subsequently the scheme is restarted.

For the discretization of the system of equations from Eq. (1c), a moving-grid finite-element method is used (Madzvamuse et al. 2003) together with the backward Euler time-integration method. The derivation of the finite-element approximation of the solution for the primary model variables is presented in Appendix 2. The integrals over the interior of the elements are approximated by a second-order accurate Newton-Cotes quadrature rule and the integrals over the boundaries of the elements are approximated by a second-order accurate Gaussian quadrature rule. Furthermore, we applied both a semi-implicit flux-corrected transport (FCT) limiter (Möller et al. 2008) and Patankar’s source / sink separation technique (Patankar 1980) on the discretized system of equations that describes the dynamics of the modeled constituents of the dermal layer. Taken together, these latter two techniques enforce positivity of the approximations of the solutions for the constituents of the dermal layer.

In order to obtain approximate solutions for the resulting systems of linear algebraic equations, we used MATLAB’s backslash operator (MathWorks 2014) after application of the LU-factorization algorithm (Davis and Duff 1997) on scaled and permuted versions of the original linear systems. For the scaling and permutation of the linear systems, several inbuilt scaling and permutation routines of MATLAB were used (Davis et al. 2004; Duff and Koster 1999) together with the permutation routine HSL_MC64 (HSL 2013).

The individual time steps were chosen automatically by using an automatically adaptive time-stepping method with inbuilt local truncation error control (Kavetski et al. 2002). The maximum size of the initial time step is set to $10^{-5}$ dimensionless units and the upper bound of the size of the time step is set to $10^{-3}$ dimensionless units. If a time step is accepted, then the subsequent time step is at most 1.25 times the size of the current time step. If a time step is rejected, then the subsequent time step is at least a quarter of the size of the current time step. For completeness, we mention here also that we set the absolute and relative truncation error tolerance to, respectively, $10^{-2}$ and $5\times 10^{-2}$ dimensionless units. (See the article by Kavetski et al. (2001) for further details.) After obtaining and accepting an approximation for a certain time step, the local extrapolation procedure proposed by Kavetski et al. (2002) is applied to increase the accuracy of the approximation.

Finally, the element resolution refinement / recoarsement tool is applied after every ten time steps in order to adjust the resolution of the elements of the triangulation. For this end, the $L_{2}$-norm of an estimation of the error in the gradient of the numerical approximation of the solution for the concentration of the collagen molecules per element is determined first (Möller and Kuzmin 2006). Subsequently, in order to adjust the estimated error, the resolution of the elements is adapted in a fashion identical to the procedure described above for the adaptation of the resolution of the elements of the base triangulation. For the interpolation of the approximations to new vertices in the triangulation, we used a piecewise bivariate Hermite interpolation (Feng and Zhang 2013). The required gradients of the approximations at the existing vertices were estimated by using a polynomial preserving gradient recovery scheme (Zhang and Naga 2005).

## Appendix 2: Derivation of the general finite-element approximation

In order to derive the general finite-element approximation, we rewrite Eq. (1b) as a systems of equations, using the assumptions described in Sect. 2.5:  where

41 $$\begin{aligned} \mathbf {\sigma } = \begin{bmatrix} \sigma _{11}&\sigma _{12}&\sigma _{13} \\ \sigma _{21}&\sigma _{22}&\sigma _{23} \\ \sigma _{31}&\sigma _{32}&\sigma _{33} \end{bmatrix} = \begin{bmatrix}&\\ \sigma _{\cdot 1}&\sigma _{\cdot 2}&\sigma _{\cdot 3} \\&\end{bmatrix}, \quad \text {and}\quad \mathbf {f} = \begin{bmatrix} f_{1} \\ f_{2} \\ f_{3} \end{bmatrix}.\nonumber \\ \end{aligned}$$

We also rewrite Eq. (1c) as a system of equations, using again the assumptions described in Sect. 2.5. Furthermore, we add the term $\varepsilon _{ij}[\nabla \cdot \mathbf {v}]$ for $i,j \in \{2,3\}$ to the left hand side (LHS) and the right hand side (RHS) of these evolution equations. That is, to the evolution equation that describes the dynamic change of $\varepsilon _{22}$ we add the term $\varepsilon _{22}[\nabla \cdot \mathbf {v}]$ to the LHS and the RHS, to the evolution equation that describes the dynamic change of $\varepsilon _{23}$ we add the term $\varepsilon _{23}[\nabla \cdot \mathbf {v}]$ to the LHS and the RHS, and so on. Finally, we rewrite the individual equations of the system and make use of the fact that the effective strain tensor is symmetric for all time *t*, and obtain:  where

47 $$\begin{aligned} \mathbf {G} = \begin{bmatrix} G_{11}&G_{12}&G_{13} \\ G_{21}&G_{22}&G_{23} \\ G_{31}&G_{32}&G_{33} \end{bmatrix} = \begin{bmatrix} G_{11}&G_{21}&G_{31} \\ G_{21}&G_{22}&G_{32} \\ G_{31}&G_{32}&G_{33} \end{bmatrix}. \end{aligned}$$

Next, we derive the weak formulations of Eqs. (1a), (39), and (40). For this end, we multiply these equations by a test function $\eta (\mathbf {x},t) \in H^{1}(\Omega _{\mathbf {x},t}),\ t \in [0,T]$, where *T* is the total simulation time, first. Subsequently, we integrate the equations over the domain of computation and apply Green’s first identity. Taken together this results in the following:

48 $$\begin{aligned}&\int \limits _{\Omega _{\mathbf {x},t}}\left\{ \eta \frac{\text {D}z_{i}}{\text {D}t} + \eta z_{i}[\nabla \cdot \mathbf {v}]\right\} \mathrm {d}\Omega _{\mathbf {x},t} \nonumber \\&= \int \limits _{\Omega _{\mathbf {x},t}}\left[ \nabla \eta \cdot \mathbf {J}_{i} + \eta R_{i}\right] \mathrm {d}\Omega _{\mathbf {x},t} - \int \limits _{\partial \Omega _{\mathbf {x},t}}\eta \left[ \mathbf {J}_{i}\cdot \mathbf {n}\right] \mathrm {d}\Gamma _{\mathbf {x},t}, \end{aligned}$$

49 $$\begin{aligned}&\rho _{t}\int \limits _{\Omega _{\mathbf {x},t}}\left\{ \eta \frac{\text {D}v_{2}}{\text {D}t} + \eta v_{2}[\nabla \cdot \mathbf {v}]\right\} \mathrm {d}\Omega _{\mathbf {x},t} \nonumber \\&= \int \limits _{\Omega _{\mathbf {x},t}}\left[ \eta f_{2} - \nabla \eta \cdot \sigma _{\cdot 2}\right] \mathrm {d}\Omega _{\mathbf {x},t} +\int \limits _{\partial \Omega _{\mathbf {x},t}}\eta \left[ \sigma _{\cdot 2}\cdot \mathbf {n}\right] \mathrm {d}\Gamma _{\mathbf {x},t}, \end{aligned}$$

50 $$\begin{aligned}&\rho _{t}\int \limits _{\Omega _{\mathbf {x},t}}\left\{ \eta \frac{\text {D}v_{3}}{\text {D}t} + \eta v_{3}[\nabla \cdot \mathbf {v}]\right\} \mathrm {d}\Omega _{\mathbf {x},t} \nonumber \\&= \int \limits _{\Omega _{\mathbf {x},t}}\left[ \eta f_{3} - \nabla \eta \cdot \sigma _{\cdot 3}\right] \mathrm {d}\Omega _{\mathbf {x},t} +\int \limits _{\partial \Omega _{\mathbf {x},t}}\eta \left[ \sigma _{\cdot 3}\cdot \mathbf {n}\right] \mathrm {d}\Gamma _{\mathbf {x},t}, \end{aligned}$$

with $\mathbf {n}$ the unit outward pointing normal vector to the boundary of the domain of computation. Next, we apply the product rule of differentiation on the first term of the integrands on the LHS of the above equations and we use Reynold’s transport theorem on the LHS of the above equations. Taken together this results in the following weak formulations. Find $z_{i}(\mathbf {x},t) \in H^{1}(\Omega _{\mathbf {x},t}),\ t \in [0,T]$ for $i \in \{N,M,c,\rho \}, v_{2}(\mathbf {x},t) \in H^{1}(\Omega _{\mathbf {x},t}),\ t \in [0,T]$, and $v_{3}(\mathbf {x},t) \in H^{1}(\Omega _{\mathbf {x},t}),\ t \in [0,T]$ such that

51 $$\begin{aligned}&\frac{\mathrm {d}}{\mathrm {d}t}\int \limits _{\Omega _{\mathbf {x},t}}\eta z_{i}\mathrm {d}\Omega _{\mathbf {x},t} = \int \limits _{\Omega _{\mathbf {x},t}}\left[ \nabla \eta \cdot \mathbf {J}_{i} + \eta R_{i} + z_{i}\frac{\text {D}\eta }{\text {D}t}\right] \mathrm {d}\Omega _{\mathbf {x},t}\nonumber \\&\quad -\int \limits _{\partial \Omega _{\mathbf {x},t}}\eta \left[ \mathbf {J}_{i}\cdot \mathbf {n}\right] \mathrm {d}\Gamma _{\mathbf {x},t}, \end{aligned}$$

52 $$\begin{aligned}&\rho _{t}\frac{\mathrm {d}}{\mathrm {d}t}\int \limits _{\Omega _{\mathbf {x},t}}\eta v_{2}\mathrm {d}\Omega _{\mathbf {x},t} = \int \limits _{\Omega _{\mathbf {x},t}}\left[ \eta f_{2} - \nabla \eta \cdot \sigma _{\cdot 2} + v_{2}\frac{\text {D}\eta }{\text {D}t}\right] \mathrm {d}\Omega _{\mathbf {x},t}\nonumber \\&\quad +\int \limits _{\partial \Omega _{\mathbf {x},t}}\eta \left[ \sigma _{\cdot 2}\cdot \mathbf {n}\right] \mathrm {d}\Gamma _{\mathbf {x},t}, \end{aligned}$$

53 $$\begin{aligned}&\rho _{t}\frac{\mathrm {d}}{\mathrm {d}t}\int \limits _{\Omega _{\mathbf {x},t}}\eta v_{3}\mathrm {d}\Omega _{\mathbf {x},t} = \int \limits _{\Omega _{\mathbf {x},t}}\left[ \eta f_{3} - \nabla \eta \cdot \sigma _{\cdot 3} + v_{3}\frac{\text {D}\eta }{\text {D}t}\right] \mathrm {d}\Omega _{\mathbf {x},t}\nonumber \\&\quad +\int \limits _{\partial \Omega _{\mathbf {x},t}}\eta \left[ \sigma _{\cdot 3}\cdot \mathbf {n}\right] \mathrm {d}\Gamma _{\mathbf {x},t}, \end{aligned}$$

for all $$\eta (\mathbf {x},t) \in H^{1}(\Omega _{\mathbf {x},t}),\ t \in [0,T]$$.

Repeating the above procedure without the application of Green’s first identity results in the following weak formulations of Eqs. (43), (44), and (45). Find $\varepsilon _{22}(\mathbf {x},t) \in L^{2}(\Omega _{\mathbf {x},t}),\ t \in [0,T], \varepsilon _{23}(\mathbf {x},t) \in L^{2}(\Omega _{\mathbf {x},t}),\ t \in [0,T]$, and $\varepsilon _{33}(\mathbf {x},t) \in L^{2}(\Omega _{\mathbf {x},t}),\ t \in [0,T]$ such that

54 $$\begin{aligned}&\frac{\mathrm {d}}{\mathrm {d}t}\int \limits _{\Omega _{\mathbf {x},t}}\eta \varepsilon _{22}\mathrm {d}\Omega _{\mathbf {x},t}\nonumber \\&\quad = \int \limits _{\Omega _{\mathbf {x},t}}\left\{ \varepsilon _{22}\frac{\text {D}\eta }{\text {D}t} - \eta \left\{ [\varepsilon _{33} - 1]\frac{\partial v_{2}}{\partial y} + G_{22}\right\} \right\} \mathrm {d}\Omega _{\mathbf {x},t} \nonumber \\&\qquad +\int \limits _{\Omega _{\mathbf {x},t}}\eta \left\{ \varepsilon _{22}\frac{\partial v_{3}}{\partial z} + \frac{1}{2}\left[ \frac{\partial v_{2}}{\partial z} - \frac{\partial v_{3}}{\partial y}\right] \left[ \varepsilon _{23} + \varepsilon _{32}\right] \right\} \mathrm {d}\Omega _{\mathbf {x},t}, \nonumber \\\end{aligned}$$

55 $$\begin{aligned}&\frac{\mathrm {d}}{\mathrm {d}t}\int \limits _{\Omega _{\mathbf {x},t}}\eta \varepsilon _{23}\mathrm {d}\Omega _{\mathbf {x},t} \nonumber \\&\quad =\int \limits _{\Omega _{\mathbf {x},t}}\left\{ \varepsilon _{23}\frac{\text {D}\eta }{\text {D}t} - \eta \left\{ G_{23} - \varepsilon _{23}\left[ \frac{\partial v_{2}}{\partial y} + \frac{\partial v_{3}}{\partial z}\right] \right\} \right\} \mathrm {d}\Omega _{\mathbf {x},t} \nonumber \\&\qquad +\frac{1}{2}\int \limits _{\Omega _{\mathbf {x},t}}\eta \left\{ [1 - 2\varepsilon _{22}]\frac{\partial v_{2}}{\partial z} + [1 - 2\varepsilon _{33}]\frac{\partial v_{3}}{\partial y}\right\} \mathrm {d}\Omega _{\mathbf {x},t}, \nonumber \\\end{aligned}$$

56 $$\begin{aligned}&\frac{\mathrm {d}}{\mathrm {d}t}\int \limits _{\Omega _{\mathbf {x},t}}\eta \varepsilon _{33}\mathrm {d}\Omega _{\mathbf {x},t} \nonumber \\&\quad = \int \limits _{\Omega _{\mathbf {x},t}}\left\{ \varepsilon _{33}\frac{\text {D}\eta }{\text {D}t} - \eta \left\{ [\varepsilon _{22} - 1]\frac{\partial v_{3}}{\partial z} + G_{33}\right\} \right\} \mathrm {d}\Omega _{\mathbf {x},t} \nonumber \\&\qquad +\int \limits _{\Omega _{\mathbf {x},t}}\eta \left\{ \varepsilon _{33}\frac{\partial v_{2}}{\partial y} - \frac{1}{2}\left[ \frac{\partial v_{2}}{\partial z} - \frac{\partial v_{3}}{\partial y}\right] \left[ \varepsilon _{23} + \varepsilon _{32}\right] \right\} \mathrm {d}\Omega _{\mathbf {x},t},\nonumber \\ \end{aligned}$$

for all $$\eta (\mathbf {x},t) \in L^{2}(\Omega _{\mathbf {x},t}),\ t \in [0,T]$$.

In order to derive the finite-element approximation, we give the general triangulation of the domain of computation $\Omega _{\mathbf {x},t}$ first. Subsequently, we introduce the used finite-dimensional subspace of the Hilbert space $H^{1}(\Omega _{\mathbf {x},t}),\ t \in [0,T]$ and the Lebesgue space $L^{2}(\Omega _{\mathbf {x},t}),\ t \in [0,T]$, and then we introduce the used basis (shape) functions for this finite-dimensional subspace. Finally, we present new versions of the above derived weak formulations. These new versions of the weak formulations are the general finite-element approximations of the solution for the primary model variables from Eq. (1c).

In this study, we used the following general triangulation $\mathcal {T}_{h,t}$ of the domain of computation $\Omega _{\mathbf {x},t},\ t \in [0,T]$:

57 $$\begin{aligned} \Omega _{\mathbf {x},t} = \bigcup _{K_{t} \in \mathcal {T}_{h,t}}K_{t}, \end{aligned}$$

where

- the elements $K_{t}$ of the triangulation are straight triangles with the general property that $\text {meas}(K_{t})\ne 0$,
- $\text {meas}(K_{t}^{1} \cap K_{t}^{2}) = 0$ for every pair of distinct elements $K_{t}^{1},K_{t}^{2} \in \mathcal {T}_{h,t}$,
- if $F = K_{t}^{1} \cap K_{t}^{2}\ne \emptyset$, then *F* is either a common side or a common vertex of $K_{t}^{1}$ and $K_{t}^{2}$,
- $$\text {diam}(K_{t}) \le h$$ for all $$K_{t} \in \mathcal {T}_{h,t}$$.

Each of the elements $K_{t}$ of $\mathcal {T}_{h,t}$ is obtained by applying the following invertible affine mapping $T_{K,t}$ on a reference triangle $\hat{K}$ with vertices (0, 0), (1, 0), and (0, 1) through $K_{t} = T_{K,t}(\hat{K})$ with $T_{K,t}$ defined by

58 $$\begin{aligned} T_{K,t}(\hat{\mathbf {x}}) = \mathbf {B}_{K,t}\hat{\mathbf {x}} + \mathbf {b}_{K,t}. \end{aligned}$$

Here, the coordinates $\hat{\mathbf {x}}$ are the reference coordinates.

Let us now introduce the used finite-dimensional subspace that has been used in this study:

59 $$\begin{aligned} X_{h}(t) = \left\{ \eta ^{h}\in C^{0}(\overline{\Omega }_{\mathbf {x},t})\ |\ \hat{\eta }_{K}^{h} \in \mathbb {P}_{1}\ \forall K_{t} \in \mathcal {T}_{h,t},\ t \in [0,T]\right\} , \end{aligned}$$

with $\eta ^{h}(\mathbf {x},t) = \hat{\eta }_{K}^{h}(T_{K,t}^{-1}(\mathbf {x}))$ and $\mathbb {P}_{1}$ the space of polynomials of degree less than or equal to one in two space variables defined on the reference triangle $\hat{K}$. (See for example Quarteroni and Valli (2008) for a proof of the fact that $X_{h}(t) \subset H^{1}(\Omega _{\mathbf {x},t}),\ t \in [0,T]$, and see the Sobolev embedding theorem for a proof of the fact that $H^{1}(\Omega _{\mathbf {x},t}),\ t \in [0,T] \subset L^{2}(\Omega _{\mathbf {x},t}),\ t \in [0,T]$ (Quarteroni and Valli 2008). Note that these inclusions also imply $X_{h}(t) \subset L^{2}(\Omega _{\mathbf {x},t}),\ t \in [0,T]$.)

In this study, the values of the functions $\eta _{h}(\mathbf {x},t)$ at the vertices of each element $K_{t}$ have been chosen as the degrees of freedom on each of these elements. Denoting by $N_{h}$ the total number of vertices in the triangulation $\mathcal {T}_{h,t}$ and denoting by $\mathbf {a}_{j},\ j \in \{1,\ldots ,N_{h}\}$ the coordinates of these vertices within $\overline{\Omega }_{\mathbf {x},t}$, we chose the functions $\varphi _{i} \in X_{h}(t)$ with

60 $$\begin{aligned} \varphi _{i}(\mathbf {a}_{j},t) = \delta _{ij},\quad i,j\in \{1,\ldots ,N_{h}\} \end{aligned}$$

as basis (shape) functions for the finite-dimensional subspace $X_{h}(t)$.

Hence, we are looking for finite-element approximations $z_{i}^{h}(\mathbf {x},t), v_{2}^{h}(\mathbf {x},t), v_{3}^{h}(\mathbf {x},t), \varepsilon _{22}^{h}(\mathbf {x},t), \varepsilon _{23}^{h}(\mathbf {x},t), \varepsilon _{33}^{h}(\mathbf {x},t) \in X_{h}(t), t \in [0,T]$ for $i \in \{N,M,c,\rho \}$ with

61 $$\begin{aligned}&z_{i}^{h}(\mathbf {x},t)= \sum _{j=1}^{N_{h}}z_{i}^{j}(t)\varphi _{j}(\mathbf {x},t),\nonumber \\&v_{2}^{h}(\mathbf {x},t) = \sum _{j=1}^{N_{h}}v_{2}^{j}(t)\varphi _{j}(\mathbf {x},t),\nonumber \\&v_{3}^{h}(\mathbf {x},t)= \sum _{j=1}^{N_{h}}v_{3}^{j}(t)\varphi _{j}(\mathbf {x},t),\nonumber \\&\varepsilon _{22}^{h}(\mathbf {x},t) = \sum _{j=1}^{N_{h}}\varepsilon _{22}^{j}(t)\varphi _{j}(\mathbf {x},t), \nonumber \\&\varepsilon _{23}^{h}(\mathbf {x},t) = \sum _{j=1}^{N_{h}}\varepsilon _{23}^{j}(t)\varphi _{j}(\mathbf {x},t),\nonumber \\&\varepsilon _{33}^{h}(\mathbf {x},t) = \sum _{j=1}^{N_{h}}\varepsilon _{33}^{j}(t)\varphi _{j}(\mathbf {x},t), \end{aligned}$$

such that

62 $$\begin{aligned}&\frac{\mathrm {d}}{\mathrm {d}t}\int \limits _{\Omega _{\mathbf {x},t}}\eta ^{h}z_{i}^{h}\mathrm {d}\Omega _{\mathbf {x},t} \nonumber \\&\quad =\int \limits _{\Omega _{\mathbf {x},t}}\left[ \nabla \eta ^{h}\cdot \mathbf {J}_{i}^{h} + \eta {h} R_{i}^{h} + z_{i}^{h}\frac{\text {D}\eta ^{h}}{\text {D}t}\right] \mathrm {d}\Omega _{\mathbf {x},t}\nonumber \\&\qquad -\int \limits _{\partial \Omega _{\mathbf {x},t}}\eta ^{h}\left[ \mathbf {J}_{i}^{h}\cdot \mathbf {n}\right] \mathrm {d}\Gamma _{\mathbf {x},t}, \end{aligned}$$

63 $$\begin{aligned}&\rho _{t}\frac{\mathrm {d}}{\mathrm {d}t}\int \limits _{\Omega _{\mathbf {x},t}}\eta ^{h}v_{2}^{h}\mathrm {d}\Omega _{\mathbf {x},t} \nonumber \\&\quad =\int \limits _{\Omega _{\mathbf {x},t}}\left[ \eta ^{h}f_{2}^{h} - \nabla \eta ^{h}\cdot \sigma _{\cdot 2}^{h} + v_{2}^{h}\frac{\text {D}\eta ^{h}}{\text {D}t}\right] \mathrm {d}\Omega _{\mathbf {x},t}\nonumber \\&\qquad +\int \limits _{\partial \Omega _{\mathbf {x},t}}\eta ^{h}\left[ \sigma _{\cdot 2}^{h}\cdot \mathbf {n}\right] \mathrm {d}\Gamma _{\mathbf {x},t}, \end{aligned}$$

64 $$\begin{aligned}&\rho _{t}\frac{\mathrm {d}}{\mathrm {d}t}\int \limits _{\Omega _{\mathbf {x},t}}\eta ^{h}v_{3}^{h}\mathrm {d}\Omega _{\mathbf {x},t} \nonumber \\&\quad =\int \limits _{\Omega _{\mathbf {x},t}}\left[ \eta ^{h}f_{3}^{h} - \nabla \eta ^{h}\cdot \sigma _{\cdot 3}^{h} + v_{3}^{h}\frac{\text {D}\eta ^{h}}{\text {D}t}\right] \mathrm {d}\Omega _{\mathbf {x},t}\nonumber \\&\qquad +\int \limits _{\partial \Omega _{\mathbf {x},t}}\eta ^{h}\left[ \sigma _{\cdot 3}^{h}\cdot \mathbf {n}\right] \mathrm {d}\Gamma _{\mathbf {x},t}, \end{aligned}$$

65 $$\begin{aligned}&\frac{\mathrm {d}}{\mathrm {d}t}\int \limits _{\Omega _{\mathbf {x},t}}\eta ^{h}\varepsilon _{22}^{h}\mathrm {d}\Omega _{\mathbf {x},t} \nonumber \\&\quad =\int \limits _{\Omega _{\mathbf {x},t}}\left\{ \varepsilon _{22}^{h}\frac{\text {D}\eta ^{h}}{\text {D}t} - \eta ^{h}\left\{ [\varepsilon _{33}^{h} - 1]\frac{\partial v_{2}^{h}}{\partial y} + G_{22}^{h}\right\} \right\} \mathrm {d}\Omega _{\mathbf {x},t} \nonumber \\&\qquad +\int \limits _{\Omega _{\mathbf {x},t}}\eta ^{h}\left\{ \varepsilon _{22}^{h}\frac{\partial v_{3}^{h}}{\partial z} + \frac{1}{2}\left[ \frac{\partial v_{2}^{h}}{\partial z} - \frac{\partial v_{3}^{h}}{\partial y}\right] \left[ \varepsilon _{23}^{h} + \varepsilon _{32}^{h}\right] \right\} \mathrm {d}\Omega _{\mathbf {x},t}, \nonumber \\\end{aligned}$$

66 $$\begin{aligned}&\frac{\mathrm {d}}{\mathrm {d}t}\int \limits _{\Omega _{\mathbf {x},t}}\eta ^{h}\varepsilon _{23}^{h}\mathrm {d}\Omega _{\mathbf {x},t} \nonumber \\&\quad =\int \limits _{\Omega _{\mathbf {x},t}}\left\{ \varepsilon _{23}^{h}\frac{\text {D}\eta ^{h}}{\text {D}t} - \eta ^{h}\left\{ G_{23}^{h} - \varepsilon _{23}^{h}\left[ \frac{\partial v_{2}^{h}}{\partial y} + \frac{\partial v_{3}^{h}}{\partial z}\right] \right\} \right\} \mathrm {d}\Omega _{\mathbf {x},t} \nonumber \\&\qquad +\frac{1}{2}\int \limits _{\Omega _{\mathbf {x},t}}\eta ^{h}\left\{ [1 - 2\varepsilon _{22}^{h}]\frac{\partial v_{2}^{h}}{\partial z} + [1 - 2\varepsilon _{33}^{h}]\frac{\partial v_{3}^{h}}{\partial y}\right\} \mathrm {d}\Omega _{\mathbf {x},t}, \nonumber \\\end{aligned}$$

67 $$\begin{aligned}&\frac{\mathrm {d}}{\mathrm {d}t}\int \limits _{\Omega _{\mathbf {x},t}}\eta ^{h}\varepsilon _{33}^{h}\mathrm {d}\Omega _{\mathbf {x},t} \nonumber \\&\quad =\int \limits _{\Omega _{\mathbf {x},t}}\left\{ \varepsilon _{33}^{h}\frac{\text {D}\eta ^{h}}{\text {D}t} - \eta ^{h}\left\{ [\varepsilon _{22}^{h} - 1]\frac{\partial v_{3}^{h}}{\partial z} + G_{33}^{h}\right\} \right\} \mathrm {d}\Omega _{\mathbf {x},t} \nonumber \\&\qquad +\int \limits _{\Omega _{\mathbf {x},t}}\eta ^{h}\left\{ \varepsilon _{33}^{h}\frac{\partial v_{2}^{h}}{\partial y} - \frac{1}{2}\left[ \frac{\partial v_{2}^{h}}{\partial z} - \frac{\partial v_{3}^{h}}{\partial y}\right] \left[ \varepsilon _{23}^{h} + \varepsilon _{32}^{h}\right] \right\} \mathrm {d}\Omega _{\mathbf {x},t},\nonumber \\ \end{aligned}$$

for all $\eta ^{h}(\mathbf {x},t) \in X_{h}(t),\ t \in [0,T]$. Here $\mathbf {J}_{i}^{h}, R_{i}^{h}, \sigma _{\cdot 2}^{h}, \sigma _{\cdot 3}^{h}, f_{2}^{h}, f_{3}^{h}, G_{22}^{h}, G_{23}^{h}$, and $G_{33}^{h}$ are equal to, respectively, $\mathbf {J}_{i}, R_{i}, \sigma _{\cdot 2}, \sigma _{\cdot 3}, f_{2}, f_{3}, G_{22}, G_{23}$, and $G_{33}$ where the primary model variables have been replaced by their respective finite-element approximations from Eq. (61).

The linear systems of algebraic equations related the above weak formulations can be obtained now by applying consecutively the first-order backward Euler method and the fixed-point defect correction method, and by replacing $\eta ^{h}(\mathbf {x},t)$ with $\varphi _{j}(\mathbf {x},t)$ for $j \in \{1,\ldots ,N_{h}\}$. (See for example Van Kan et al. (2014) for more details about these procedures.) Finally, note that the following holds for the chosen basis functions of the subspace $X_{h}(t)$ (Dziuk and Elliott 2007):

68 $$\begin{aligned} \frac{\text {D}\varphi _{j}}{\text {D}t} = 0. \end{aligned}$$

This property of the chosen basis functions simplifies the construction of the linear systems of equations.

## Appendix 3: The parameter value estimates

See Table 1.

**Table 1:** An overview of the dimensional (ranges of the) values for the parameters of the model

Parameter	Value	Dimensions	Reference
$D_{F}$	$$10^{-7}\ $$	$\text {cm}^{5}/(\text {cells day})$	Sillman et al. (2003)
$\chi _{F}$	$$2\times 10^{-3}\ $$	$\text {cm}^{5}/(\text {g day})$	Murphy et al. (2012)
*q*	$$-4.2\times 10^{-1}\ $$	−	NC
$r_{F}$	$$9.24\times 10^{-1}\ $$	$\text {cm}^{3q}/(\text {cells}^{q}\ \text {day})$	Ghosh et al. (2007)
$r_{F}^{\max }$	$$2\ $$	−	Strutz et al. (2001)
$a_{c}^{I}$	$$10^{-8}\ $$	$\text {g}/\text {cm}^{3}$	Grotendorst (1992)
$\kappa _{F}$	$$10^{-6}\ $$	$\text {cm}^{3}/\text {cells}$	Vande Berg et al. (1989)
$k_{F}$	$$1.08\times 10^{7}\ $$	$\text {cm}^{3}/(\text {g day})$	Desmoulière et al. (1993)
$\delta _{N}$	$$2\times 10^{-2}\ $$	$/\text {day}$	Olsen et al. (1995)
$\delta _{M}$	$$6\times 10^{-2}\ $$	$/\text {day}$	Koppenol et al. (2017b)
$D_{c}$	$$2.9\times 10^{-3}\ $$	$\text {cm}^{2}/\text {day}$	Murphy et al. (2012)
$k_{c}$	$$4\times 10^{-13}\ $$	$\text {g}/(\text {cells day})$	Olsen et al. (1995)
$\eta ^{I}$	$$2\ $$	−	Rudolph and Vande Berg (1991),
			Moulin et al. (1998)
$a_{c}^{II}$	$$10^{-8}\ $$	$\text {g}/\text {cm}^{3}$	Olsen et al. (1995)
$\delta _{c}$	$$5\times 10^{-4}\ $$	$\text {cm}^{6}/(\text {cells g day})$	Olsen et al. (1995)
$\eta ^{II}$	$$5\times 10^{-1}\ $$	−	TW
$a_{c}^{III}$	$$(2 - 2.5)\times 10^{8}\ $$	$\text {cm}^{3}/\text {g}$	Overall et al. (1991)
$k_{\rho }$	$$6\times 10^{-8}\ $$	$\text {g}/(\text {cells day})$	NC
$k_{\rho }^{\max }$	$$10\ $$	−	Olsen et al. (1995)
$a_{c}^{IV}$	$$10^{-9}\ $$	$\text {g}/\text {cm}^{3}$	Roberts et al. (1986)
$\delta _{\rho }$	$$6\times 10^{-6}\ $$	$\text {cm}^{6}/(\text {cells g day})$	Koppenol et al. (2017b)
$\rho _{t}$	1.02	$\text {g}/\text {cm}^{3}$	Liang and Boppart (2010)
$\mu _{1}$	$$10^{2}\ $$	$(\text {N day})/\text {cm}^{2}$	TW
$\mu _{2}$	$$10^{2}\ $$	$(\text {N day})/\text {cm}^{2}$	TW
$E^{I}$	$$3.2\times 10\ $$	$\text {N}/((\text {g cm})^{1/2})$	Liang and Boppart (2010)
$\nu$	$$4.9\times 10^{-1}\ $$	−	Liang and Boppart (2010)
$\xi$	$$5\times 10^{-2}\ $$	$(\text {N g})/(\text {cells cm}^{2})$	Maskarinec et al. (2009)
			&Wrobel et al. (2002)
*R*	$$9.95\times 10^{-1}\ $$	$\text {g}/\text {cm}^{3}$	TW
$\zeta$	$$(0 - 9)\times 10^{2}\ $$	$\text {cm}^{6}/(\text {cells g day})$	TW
$\overline{N}$	$$10^{4}\ $$	$\text {cells}/\text {cm}^{3}$	Olsen et al. (1995)
$\overline{M}$	$$0\ $$	$\text {cells}/\text {cm}^{3}$	Olsen et al. (1995)
$\overline{c}$	$$0\ $$	$\text {g}/\text {cm}^{3}$	NC
$\overline{\rho }$	$$10^{-1}\ $$	$\text {g}/\text {cm}^{3}$	Olsen et al. (1995)
$I^{w}$	$$2\times 10^{-1}\ $$	−	Koppenol et al. (2017b)
$c^{w}$	$$10^{-8}\ $$	$\text {g}/\text {cm}^{3}$	Olsen et al. (1995)

The last column contains the references to the articles that were used for obtaining (estimates of) the values for the parameters. If (the range of) the value for a parameter was estimated in this study, then this is indicated by the abbreviation TW. If the value for a parameter is a necessary consequence of the values chosen for the other parameters, then this is indicated by the abbreviation NC
